# Social Competence in Higher Education Questionnaire (CCSES): Revision and Psychometric Analysis

**DOI:** 10.3389/fpsyg.2016.01484

**Published:** 2016-10-18

**Authors:** Esther N. Leganés-Lavall, Santiago Pérez-Aldeguer

**Affiliations:** ^1^Institute for Research in the Social Sciences, Arts, and HumanitiesMadrid, Spain; ^2^Department of Music, Arts and Sports, Universidad de ZaragozaZaragoza, Spain

**Keywords:** social competence, higher education, psychometrics, professional competences, questionnaire

## Introduction

Over the past decades several researches have addressed the social competence from the university setting by its importance for education and society (Hidalgo and Abarca, 1990; Johnson et al., 1991; Oberst et al., 2009). Social factors play an outstanding role in the learning process of higher education students as they are being professionally prepared to actively participate and contribute in a changing society (Del Prette et al., 1999; Marín and León, 2001). According to Schoon (2009), an increasing globalization and use of technologies have led to a series of socio-historical changes were social competence have been affected by the emergence of new values and lifestyles. Therefore, the development of social competence has become one of the main objectives of contemporary education systems and institutions (Gedvilienė, 2012). Universities are providing indicators for generic competences to evidence their effectiveness in terms of students’ learning outcomes (Xie et al., 2014). In fact, the European Commission (2005) considers social competence an essential and one of the most important precursors of prosperity and social well-being in its Member States.

Social competence refers to display socially appropriate behaviors in different circumstances and according to the social expectations of the environment (Gresham, 1995). A socially competent person is able to optimize their social behavior depending on the available social information (Taborsky and Oliveira, 2012). This ability improves his/her interaction, social relationships (Savickas, 2005) and is based on behavioral flexibility. According to the European Parliament (2006), social competence is one of the eight key competences for lifelong learning, and refers to all behaviors that allow individuals to participate in an effective and constructive way in different environments of social and working life.

Social competence in educational settings is influenced by the learning environment where highlights the ability to communicate and cooperate with each other (Gedvilienė, 2012). Successful communication and cooperation situations include a wide range of skills and behaviors as: teamwork, problem solving, decision making, facing challenges, establishing and maintaining relationships, self-control, assertiveness, responsibility, respect, creativity, or critical thinking, among others. The development of social competence from school to university years has an outstanding importance for allowing personal growth, self-esteem, and the respect for the socially established human rights. An individual with a poorly developed social competence may find difficulties to successfully interact with the events of his/her life, demonstrate positive feelings, set goals, or devise strategies, especially in adverse situations (Del Prette et al., 1999).

In the last decade, several studies coincide in asserting on the importance of designs and evaluations of programs to develop social competence among higher education students (González and Lobato, 2008; García Rojas, 2010; Pérez-Escoda et al., 2010). Karl-Heinz and Lindner-Müller (2012) also point out the importance of developing rational instruments for the measurement of social competence, as this is the first step taken in the study for both the social competence development and its impact on other crucial education results, as well as for the psychosocial development (Schoon, 2009). However, the development and measurement of social competence in higher education seems to face a challenge as the general organization of courses does not favor group work and educational goals at university are focused on academic knowledge (Buchs and Butera, 2015).

## The Development of the Social Competence in Higher Education Questionnaire

Since the academic year 2008/2009 Pérez-Aldeguer (2013a,b, 2016) has been developing and shaping a project – an Educational Musical Theater – to enhance higher education students’ cooperation and interaction. From the beginning of the process until the final performance, students need to have a continuous face-to-face interaction and cooperation to find a consensus, to solve problems, to achieve a common goal, and to overcome challenges, among others (Pérez-Aldeguer, 2013b). After the development of the pilot project it was found that several difficulties arose between the participants. The observed evidence led to the need of measure the social competence of students and inquire about the effects of the Educational Musical Theater project. However, an exhaustive search showed that there were few instruments to measure the social competence of university students, most of these measure instruments often have a long history (dating even from the 1950s and 1960s), and not-validated measures were found in Spanish language. According to Ten Dam and Volman (2007), there are: “few instruments within the societal dimension of social competence” (p. 291) and “relatively few instruments in which social competence is operationalised from the perspective of ‘educating for citizenship’” (p. 293). On the other hand, and despite of the consensus of researchers on the importance of social competence for human development, the focus has been traditionally centered in childhood (e.g., O’Malley, 1977; Gresham, 1986), adolescence (e.g., Englund et al., 2000), and special needs (Mellard and Hazel, 1992; Gresham, 1997). This fact is evidenced in the lack of standardized measures to analyze social competence in adults. For this reason, Pérez-Aldeguer (2013b) developed and validated a questionnaire to measure social competence in higher education based on the previous measures from the Group Climate Questionnaire Short Form (MacKenzie, 1983), the Group Cohesion Evaluation Questionnaire (Glass and Benshoff, 2002) and the Social Skills Scale (Goldstein et al., 1989).

Social competence can be measured from a wide variety of methods as for example, observation, self-reports, questionnaires, scales, or interviews, but there is not a consensus about how to measure social competence. However, and as recommended by Schoon (2009) it is best to select measures for the context being addressed. CCSES (Cuestionario de Competencia Social en Educación Superior) was originally validated for the measurement of students’ social competence development before and after their participation in a Musical Theater Project (Pérez-Aldeguer, 2013b) where students had to cooperate to build a common project with educational aims. CCSES was constructed according to the perspective of social competence as a multidimensional construct with emotional, cognitive, and contextual dimensions (Sarason, 1981; Waters and Sroufe, 1983; Schneider et al., 1996) which includes interactions between individual characteristics, social demands, and characteristics of the cultural context (Schoon, 2009).

Based on an extensive review of the research literature and through a series of revisions and validation processes, there were identified three dimensions: Group Climate, Team Cohesion, and Social Skills (MacKenzie, 1983; Goldstein et al., 1989; Glass and Benshoff, 2002).

### Group Climate

Group climate refers to the “the participant’s perception of the group’s atmosphere” (Kivlighan and Angelone, 1992, p. 469). Group climate has a great influence on the performance of any team. In educational settings, group climate is a fundamental of learning environment and is related to students’ emotions, feelings, and consequently with their behaviors. As future professionals, higher education students should be able to communicate effectively with others, deal with conflicts and have the skills to solve them successfully. MacKenzie (1983) identified three main attributes of group climate: participation, avoidance, and conflict.

*Participation* indicates if members want to be part of the group, contributing to group goals and sharing personal details about their lives.

*Avoidance* takes place when members refuse to discuss important issues and depend on the facilitator for guidance.

*Conflict* arises when members of a team begin to recognize their differences and they feel anxious, distrustful, distant, and withdrawn.

### Team Cohesion

Team cohesion refers to the degree to which group members wish to remain (Shivers, 1980), e.g., the strength of ties between its members, unity, feelings of attraction of its members, and own group, as well as the degree to which members coordinate their efforts to achieve common goals (Forsyth, 1999). Social competence has itself a teamwork orientation that means to be able to work with others by establishing a successful communication and constructive behavior oriented to group development.

### Social Skills

The efforts of many researchers for establishing a consensus on the conceptualization of social skills have led to two main theoretical models (Del Prette et al., 1999). On the one hand, social skills are seen as synonymous of social competence (e.g., Caballo, 1993). On the other hand, social skills are defined in a descriptive sense to refer those behaviors related to social action, and social competence is applied to evaluate the effectiveness of social action according to the social skills (Gresham, 1986). Accordingly, in this study the concept of social competence is understood as the evaluation of those: “positive social behaviors that contribute to the onset and maintenance of positive social interactions” (La Greca, 1993, p. 288). Social skills is one of the five major components of emotional intelligence (Goleman, 1998). Then, the dimension “social skills” is related to various explicit and implicit behaviors of social action (e.g., to express and regulate emotions, to be aware of others’ feelings) and can be subdivided, including both interpersonal and intrapersonal skills (Goldstein et al., 1989):

*Interpersonal skills* are the skills that allow us to communicate and interact with others, both individually and in groups.

*Intrapersonal skills* are the skills occurring within the individual’s own mind which allows effective thought processes.

The statistically rigorous approach supported the original CCSES instrument as a valid and reliable tool to measure social competence in higher education. However, there were some areas for improvement in the instrument. On the one hand, the first version of CCSES was developed for music teacher education students rating themselves before and after their participation in a group-project. To apply the instrument in different settings of higher education, some items needed to be revised. On the other hand, the original instrument sample-to-item ratio was slightly lower than 5:1 which is the minimum recommended by some authors (Hair et al., 2006).

Therefore, there was a need to revise and validate the instrument. The purpose of this study was twofold. First, the aim was to revise the items of the questionnaire that measures students’ social competence in higher education to allow the use of the instrument in different areas of knowledge and university settings regardless of whether they have been involved in a group-project or not. Second, we aimed to analyze the reliability and validity of the revised instrument with a bigger sample-to-item ratio and three research questions were identified for this study:

1. Do the forty items in the original CCSES allow compre hensively measure social competence of undergraduate students?
2. What is the reliability evidence for a revised social competence instrument?
3. What is the validity evidence for a revised social competence instrument?

## Materials and Methods

### Participants and Procedure

A sample of 523 undergraduate students (178 male and 345 female; M age = 23.2, *SD* = 1.95) enrolled in the Bachelor of Music Education, Early Childhood Education and Primary Education, and the Master of Secondary Education from different Spanish universities participated in the study at the end of the academic year.

The study was developed in two phases. In a first step, existing measurement items were revised and some of them were reworded. Experts’ reviews with four researchers from the areas of Education, Music, Psychology, and Pedagogy were conducted to ensure that meanings of the items were kept clear and understandable. In a second phase, informed consent was granted by the participants and the questionnaire was administered by one of the authors of this study. In order to ensure the anonymity of participants’ responses only demographic data as gender and age was asked. The psychometric analysis of CCSES was performed to ensure the validity and reliability of the instrument.

### Instruments

The original version of CCSES is a 40-items questionnaire on a 4-point Likert scale consisting of three variables:

1. Group climate (12 items: 1-4-7-10-13-16-19-22-25-29-34-37) measuring the climate of relations between peers in terms of participation, avoidance, and conflict.
2. Team cohesion (9 items: 2-5-8-11-14-17-20-23-26) measuring the degree in which participants wish to belong to the group.
3. Social skills (19 items: 3-6-9-12-15-18-21-24-27-28-30-31-32-33-35-36-38-39-40) where interpersonal and intrapersonal peer interaction is assessed.

After a revision, some items were reworded for clarity (items: 3-9-10-13-32-33-39) or minor changes were introduced (items: 4-11-17- 21- 23- 26- 31). The other 26 items were kept in their original form.

### Data Analysis

In order to test the psychometric characteristics of the instrument, several analysis were performed with SPSS 17.0. First, an exploratory factor analysis (EFA) of main components was performed by applying Promax rotation with Kaiser normalization to test the construct validity of CCSES. The oblique rotation was chosen assuming the original correlations between factors. Second, the reliability or internal consistency of each variable was assessed by Cronbach’s alpha, and the correlations between variables were tested by the Pearson correlation coefficient. Finally, a confirmatory factor analysis (CFA) with three factors was conducted to test the fit between factors and items by the maximum likelihood estimation.

## Results

### Exploratory Factor Analysis

The first step was aimed to confirm the multidimensional structure of CCSES by conducting an EFA of principal components. The Measure of Sampling Adequacy Kaiser-Meyer-Olkin (0.92) and Bartlett’s test of Sphericity (χ2(877) = 7234,5; *p* < 0.00) showed values that allow the use of factor analysis and three main factors which together account for 67.47% of the variance. The first factor explains 25.20% of the total variance, and the second and third factors explain the remaining 42.27%. All 40 items were retained.

### Reliability

Cronbach’s Alpha coefficient showed a reliability of 0.89 for the overall social competence questionnaire. The coefficient alphas for the three dimensions of social competence also revealed good internal consistency. Group climate and team cohesion were above 0.80 and had improved alphas compared with the original version. Social skills and the overall coefficient of social competence had slightly decreased without compromising their reliability as the coefficients are still above 0.90. Overall internal consistency was improved in the revision (0.91) compared to the original version (0.89).

### Confirmatory Factor Analysis

**FIGURE 1:**
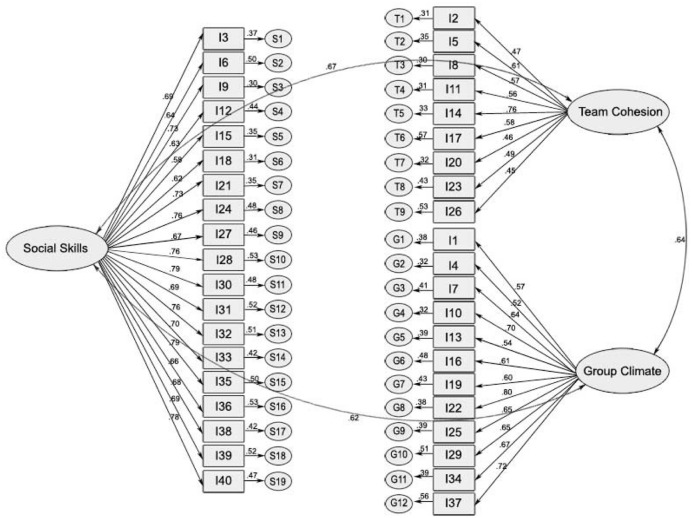
**Structural equation model (three factors model)**.

The 40 items in the three-factor model were entered for CFA. This model provided a good fit χ^2^ = 259.5, *p* < 0.001, χ^2^*/df* = 2.61, NFI = 0.92, CFI = 0.92, IFI = 0.94, and RMSEA = 0.067. All three factors (Team Cohesion, Group Climate, and Social Skills) were significantly related and factorial loadings ranged between 0.55 and 0.80 for most items as shown in **Figure 1**.

## Discussion

This study aimed to review and validate a questionnaire to measure social competence in higher education. The relevance of the study is based on the need to develop measurement instruments at University settings as noted by several researchers (Del Prette et al., 1999; González and Lobato, 2008; Oberst et al., 2009; García Rojas, 2010; Pérez-Escoda et al., 2010; Karl-Heinz and Lindner-Müller, 2012). To allow the use of the instrument in different areas of knowledge, regardless of whether they have been involved in a group-project or not, 14 items were modified after a revision. The construct validity of the instrument is well supported with a final item bank comprised of 40 items of good psychometric qualities.

The results confirmed that the CCSES questionnaire is a valid and reliable instrument to measure social competence in higher education and confirmed empirical evidence for the three-factor framework -group climate, team cohesion, and social skills- of the original instrument (Pérez-Aldeguer, 2013b). These three variables explained the 67.47% of the variance according to the responses of participants in the study. The reliability coefficients on psychometric analysis showed values that indicate good internal consistency of the 40 items together and the items on each of the factors. Specifically, the internal consistency of the factors in this study presented similar values than in previousresearch on Group Climate (0.80), Team Cohesion (0.77), and slightly higher in the Social Skills (0.92).

According to the literature on social competence (e.g., Forsyth, 1999; Gedvilienė, 2012; Buchs and Butera, 2015), this results appear of special interest since the development of group climate, team cohesion, and social skills are important variables on work-group efficacy in terms of academic outcomes, enabling and promoting the development of social competence. Moreover, there seems to be a paucity of quantitative and qualitative research on variables involved in social competence. Measuring clearly defined social competence by means of an empirically validated theoretical framework in a specific disciplinary context enables the systematic development and evaluation.

One limitation of the present study was the sample as it was composed by 523 Spanish undergraduate and postgraduate students from Education. Future studies could employ samples of students from other knowledge areas to validate the questionnaire in different areas of higher education.

Further research will be needed to extend the current studies in higher education both for development and measurement of social competence in higher education students. Nevertheless CCSES can be useful for a wide range of researchers interested in the social competence of their students.

## Conclusion

The results show that CCSES is a valid, reliable and useful instrument to measure social competence of higher education students within the Spanish context. However, to generalize the results to the entire university population, future studies should be addresses to participants from different faculties, as participants in this study come exclusively from the Education Faculty. Furthermore, alternative assessment instruments as scales of observation or interviews could be employed to complement the measurement of social competence.

## Author Contributions

All authors listed, have made substantial, direct and intellectual contribution to the work, and approved it for publication.

## Conflict of Interest Statement

The authors declare that the research was conducted in the absence of any commercial or financial relationships that could be construed as a potential conflict of interest.
