# Supplementary material for: Social Competence in Higher Education Questionnaire (CCSES): Revision and Psychometric Analysis
Source: Front Psychol. 2016 Oct 18;7:1484. doi: 10.3389/fpsyg.2016.01484 (PMC5067339; doi:10.3389/fpsyg.2016.01484)

**APPENDIX I**

**Cuestionario de Competencia Social en Educación Superior CCSES**

*Social Competence in Higher Education Questionnaire*

In the Appendix I, items are listed according to the three factors of CCSES in order to allow the reader to follow the results discussed in the article. Items are shown both in Spanish and English.

*Factor 1. Group Climate*

1. Hay buena relación entre los miembros del grupo

*There is a good relationship between the members of the group*

4. Tratamos de comprender y razonar lo que hacemos juntos

*We try to understand and reason what we do together*

7. Hablamos de los problemas que hay entre nosotros

*We talk about the problems between us*

10. Tenemos un buen sentido de participación en el grupo

*There is a good sense of participation in the group*

13. Realizamos nuestro trabajo sin depender de un líder del grupo

*We make our work without depending on a group leader*

16. Hay pocos roces y enfados entre los miembros del grupo

*There is little friction and anger among the members of the group*

19. Nos sentimos cercanos los unos a los otros

*We feel close to each other*

22. Nos esforzamos por solucionar los problemas que surgen

*We strive to solve the arising problems*

25. Actuamos conforme pensamos que es bueno para el grupo

*We act as we think that is good for the group*

29. Hay confianza entre los miembros del grupo

*There is confidence between the members of the group*

34. Entre los miembros del grupo se muestran los sentimientos abiertamente

*Our feelings are openly shown among the members of the group*

37. Los miembros del grupo estamos poco tensos y ansiosos

*The members of the group are little tense and anxious*

*Factor II. Team Cohesion*

2. Nos sentimos a gusto trabajando juntos

*We feel comfortable working together*

5. Nos sentimos bien con nuestro grupo

*We feel good with our group*

8. Disfrutamos ayudándonos los unos a los otros

*We enjoy helping each other*

11. Permanecemos juntos ante los retos

*We stay together against the challenges*

14. Me siento seguro/a en mi grupo

*I feel safe in my group*

17. Nos animamos los unos a los otros para lograr superar los retos

*We encourage each other to achieve the challenges*

20. Siento que encajo bien en el grupo

*I feel that I fit well in the group*

23. Me gustaría participar en más retos con mi grupo

*I would like to participate in more challenges with my group*

26. Nos ayudamos los unos a los otros

*We help each other*

*Factor III. Social Skills*

3. Cuando una persona me habla, presto atención y me esfuerzo por entenderla

*When somebody talks to me, I pay attention and make an effort to understand him/her*

6. Cuando no entiendo algo se lo pido a la persona adecuada

*When I do not understand something I ask to the right person*

9. Demuestro mi agradecimiento con los miembros del grupo

*I show my gratitude to the members of the group*

12. Pido ayuda cuando la necesito

*I ask for help when I need it*

15. Explico las cosas de forma que los demás pueden entenderlo fácilmente

*I explain things in a way that others can easily understand*

18. Pido perdón a los demás cuando hago algo que sé que está mal

*I apologize to others when I do something that I know is wrong*

21. Intento comprender y conocer las emociones que siento

*I try to understand the emotions that I feel*

24. Permito que los demás sepan lo que siento

*I let others to know what I feel*

27. Intento comprender lo que sienten los demás

*I try to understand what others feel*

28. Ayudo a quien lo necesita

*I help those in need*

30. Si no estoy de acuerdo con alguien trato de llegar a un acuerdo

*If I do not agree with someone, I try to reach an agreement*

31. Encuentro otras formas de resolver situaciones difíciles sin tener que enfadarme

*I find other ways to solve difficult situations without getting angry*

32. Escucho a quién intenta convencerme de algo y después decido qué hacer

*When somebody tries to convince me I listen to him/her and then I decide what to do*

33. Cuándo me acusan de algo, entiendo de qué y por qué lo hacen, y después pienso en la mejor forma de relacionarme con la persona que me acusó

*When someone accuse me of something, I understand what and why and then I think about the best way to interact with the person who accused me*

35. Pienso en la mejor forma de dar mi punto de vista antes de una conversación difícil

*I think the best way to give my point of view before a difficult conversation*

36. Si surge un problema, intento determinar qué lo causó

*If a problem arises I try to determine what caused it*

38. Me fijo un objetivo antes de empezar una tarea

*I fix an objective before starting a task*

39. Elijo con sinceridad si podría hacer bien una tarea específica antes de comenzar a realizarla

*I honestly choose if I could perform well a specific task before starting doing it*

40. Decido lo que necesito saber y cómo conseguir esa información

*I decide what I need to know and how to get that information*

**APPENDIX II**

**Cuestionario de Competencia Social en Educación Superior CCSES**

In the Appendix II, the questionnaire is shown including the Likert scale, instructions and the rating criteria.

Instrucciones: Por favor, señala con un círculo tu nivel de acuerdo o desacuerdo con cada una de las siguientes afirmaciones sobre ti y tu grupo de compañeros/as de clase.

1. Totalmente en desacuerdo
2. En desacuerdo
3. De acuerdo
4. Totalmente de acuerdo

| 1. Hay buena relación entre los miembros del grupo | 1 2 3 4 |
| --- | --- |
| 1. Nos sentimos a gusto trabajando juntos | 1 2 3 4 |
| 1. Cuando una persona me habla, presto atención y me esfuerzo por entenderla | 1 2 3 4 |
| 1. Tratamos de comprender y razonar lo que hacemos juntos | 1 2 3 4 |
| 1. Nos sentimos bien con nuestro grupo | 1 2 3 4 |
| 1. Cuando no entiendo algo se lo pido a la persona adecuada | 1 2 3 4 |
| 1. Hablamos de los problemas que hay entre nosotros | 1 2 3 4 |
| 1. Disfrutamos ayudándonos los unos a los otros | 1 2 3 4 |
| 1. Demuestro mi agradecimiento con los miembros del grupo | 1 2 3 4 |
| 1. Tenemos un buen sentido de participación en el grupo | 1 2 3 4 |
| 1. Permanecemos juntos ante los retos | 1 2 3 4 |
| 1. Pido ayuda cuando la necesito | 1 2 3 4 |
| 1. Realizamos nuestro trabajo sin depender de un líder del grupo | 1 2 3 4 |
| 1. Me siento seguro/a en mi grupo | 1 2 3 4 |
| 1. Explico las cosas de forma que los demás pueden entenderlo fácilmente | 1 2 3 4 |
| 1. Hay pocos roces y enfados entre los miembros del grupo | 1 2 3 4 |
| 1. Nos animamos los unos a los otros para lograr superar los retos | 1 2 3 4 |
| 1. Pido perdón a los demás cuando hago algo que sé que está mal | 1 2 3 4 |
| 1. Nos sentimos cercanos los unos a los otros | 1 2 3 4 |
| 1. Siento que encajo bien en el grupo | 1 2 3 4 |
| 1. Intento comprender las emociones que siento | 1 2 3 4 |
| 1. Nos esforzamos por solucionar los problemas que surgen | 1 2 3 4 |
| 1. Me gustaría participar en más retos con mi grupo | 1 2 3 4 |
| 1. Permito que los demás sepan lo que siento | 1 2 3 4 |
| 1. Actuamos conforme pensamos que es bueno para el grupo | 1 2 3 4 |
| 1. Nos ayudamos los unos a los otros | 1 2 3 4 |
| 1. Intento comprender lo que sienten los demás | 1 2 3 4 |
| 1. Ayudo a quien lo necesita | 1 2 3 4 |
| 1. Hay confianza entre los miembros del grupo | 1 2 3 4 |
| 1. Si no estoy de acuerdo con alguien. trato de llegar a un acuerdo | 1 2 3 4 |
| 1. Encuentro otras formas de resolver situaciones difíciles sin tener que enfadarme | 1 2 3 4 |
| 1. Escucho a quién intenta convencerme de algo y después decido qué hacer | 1 2 3 4 |
| 1. Cuándo me acusan de algo, entiendo de qué y por qué lo hacen, y después pienso en la mejor forma de relacionarme con la persona que me acusó | 1 2 3 4 |
| 1. Entre los miembros del grupo se muestran los sentimientos abiertamente | 1 2 3 4 |
| 1. Pienso en la mejor forma de dar mi punto de vista antes de una conversación difícil | 1 2 3 4 |
| 1. Si surge un problema, intento determinar qué lo causó | 1 2 3 4 |
| 1. Los miembros del grupo estamos poco tensos y ansiosos | 1 2 3 4 |
| 1. Me fijo un objetivo antes de empezar una tarea | 1 2 3 4 |
| 1. Elijo con sinceridad si podría hacer bien una tarea específica antes de comenzar a realizarla | 1 2 3 4 |
| 1. Decido lo que necesito saber y cómo conseguir esa información | 1 2 3 4 |

Factors: Group climate (ítems 1-4-7-10-13-16-19-22-25-29-34-37); Team cohesion (2-5-8-11-14-17-20-23-26); Social skills (3-6-9-12-15-18-21-24-27-28-30-31-32-33-35-36-38-39-40).

*Rating criteria*

Sum the total score by giving one, two, three or four points according to the responses in the Likert scale (1=1 point; 2=2 points; 3=3 points; 4=4 points).

The total composite score of the 40 items of the instrument (Social Competence) ranges from 40 to 160 points. The Group Climate (Factor 1) rates between 12 and 48, the Team Cohesion (Factor 2) is between 9 and 36, and the Social Skills (Factor 3) ranges from 19 to 76.

*Standards for interpretation of percentiles*

|  |  | Factors | | |  |
| --- | --- | --- | --- | --- | --- |
| Percentil |  | 1 | 2 | 3 | Total |
| 5 | Very low | 30 | 20 | 45 | 99 |
| 25 | Low | 33 | 24 | 55 | 112 |
| 50 | Average | 36 | 26 | 58 | 119 |
| 75 | High | 38 | 28 | 61 | 126 |
| 95 | Very High | 41 | 31 | 65 | 134 |

*
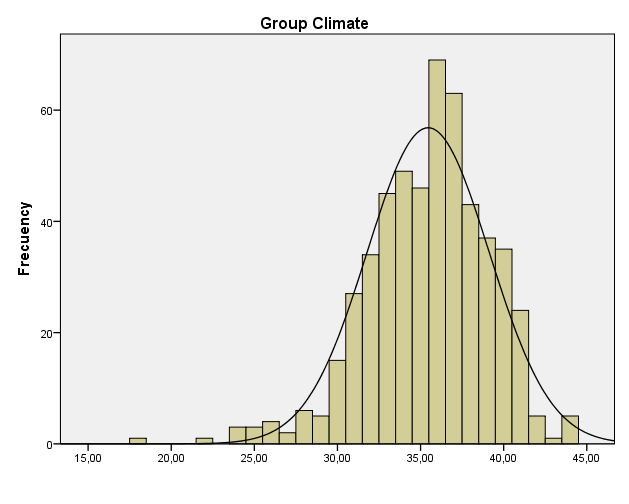

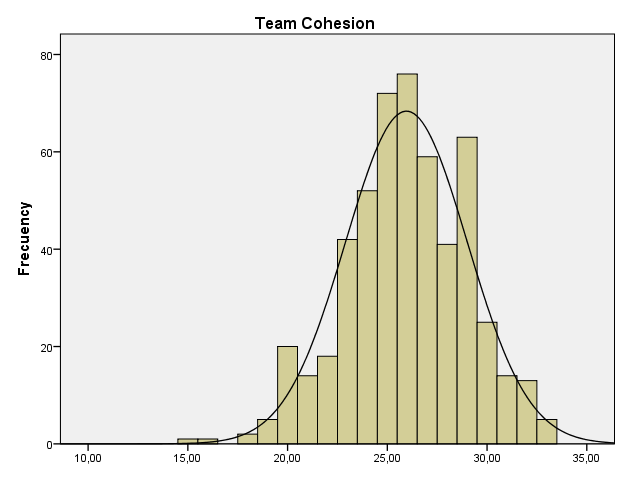
*


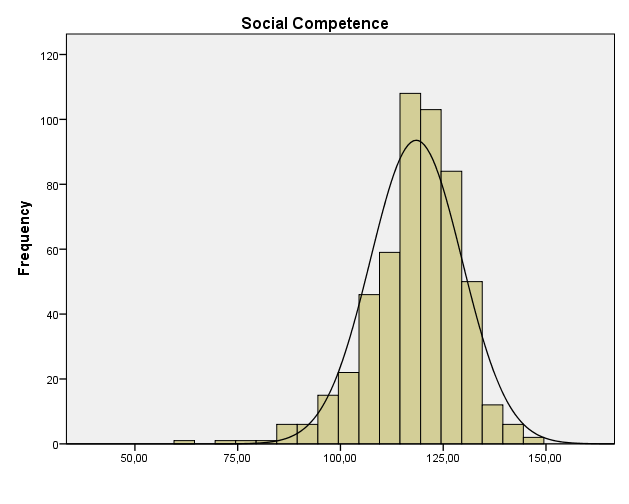

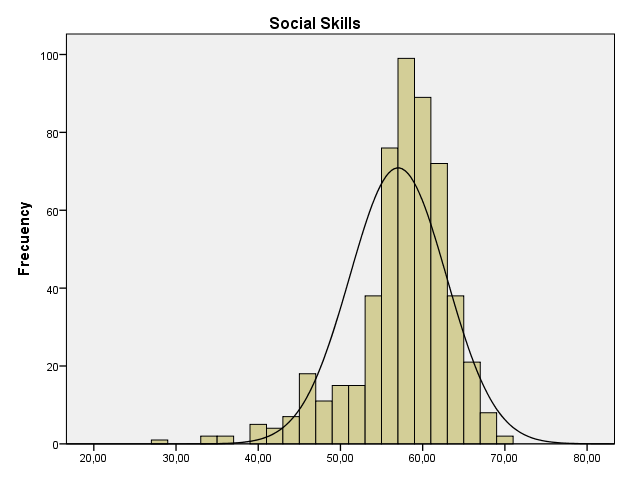

Supplement: Supplementary file 1 [file Data_Sheet_1.docx]
